# Resequencing of *Treponema pallidum* ssp. *pallidum* Strains Nichols and SS14: Correction of Sequencing Errors Resulted in Increased Separation of Syphilis Treponeme Subclusters

**DOI:** 10.1371/journal.pone.0074319

**Published:** 2013-09-10

**Authors:** Helena Pětrošová, Petra Pospíšilová, Michal Strouhal, Darina Čejková, Marie Zobaníková, Lenka Mikalová, Erica Sodergren, George M. Weinstock, David Šmajs

**Affiliations:** 1 Department of Biology, Faculty of Medicine, Masaryk University, Brno, Czech Republic; 2 Human Genome Sequencing Center, Baylor College of Medicine, Houston, Texas, United States of America; 3 Department of Genetics, The Genome Institute, Washington University School of Medicine, St. Louis, Missouri, United States of America; University of North Texas, United States of America

## Abstract

**Background:**

*Treponema pallidum* ssp. *pallidum* (TPA), the causative agent of syphilis, is a highly clonal bacterium showing minimal genetic variability in the genome sequence of individual strains. Nevertheless, genetically characterized syphilis strains can be clearly divided into two groups, Nichols-like strains and SS14-like strains. TPA Nichols and SS14 strains were completely sequenced in 1998 and 2008, respectively. Since publication of their complete genome sequences, a number of sequencing errors in each genome have been reported. Therefore, we have resequenced TPA Nichols and SS14 strains using next-generation sequencing techniques.

**Methodology/Principal Findings:**

The genomes of TPA strains Nichols and SS14 were resequenced using the 454 and Illumina sequencing methods that have a combined average coverage higher than 90x. In the TPA strain Nichols genome, 134 errors were identified (25 substitutions and 109 indels), and 102 of them affected protein sequences. In the TPA SS14 genome, a total of 191 errors were identified (85 substitutions and 106 indels) and 136 of them affected protein sequences. A set of new intrastrain heterogenic regions in the TPA SS14 genome were identified including the *tprD* gene, where both *tprD* and *tprD2* alleles were found. The resequenced genomes of both TPA Nichols and SS14 strains clustered more closely with related strains (i.e. strains belonging to same syphilis treponeme subcluster). At the same time, groups of Nichols-like and SS14-like strains were found to be more distantly related.

**Conclusion/Significance:**

We identified errors in 11.5% of all annotated genes and, after correction, we found a significant impact on the predicted proteomes of both Nichols and SS14 strains. Corrections of these errors resulted in protein elongations, truncations, fusions and indels in more than 11% of all annotated proteins. Moreover, it became more evident that syphilis is caused by treponemes belonging to two separate genetic subclusters.

## Introduction

*Treponema pallidum* ssp. *pallidum* (TPA) causes syphilis, a globally occurring venereal disease. Since TPA cannot be continuously cultivated under *in vitro* conditions, genomic data serves as a valuable source for identification of treponemal virulence factors, targets for molecular typing and candidates for potential vaccine development [1]–[4]. Moreover, characterization of genome sequences might help to define differences in pathogenicity between TPA and other pathogenic treponemes and to reconstruct treponemal evolution events [5]–[8].

Whole genome sequences of TPA strains Nichols and SS14 were published in 1998 [8] and 2008 [9], respectively. TPA strain Nichols was isolated in 1912 from the cerebrospinal fluid of syphilitic patient in Washington D.C. [10], and since that time it has been propagated in rabbits and used as a reference laboratory strain. TPA strain SS14 was isolated in 1977 in Atlanta, GA, from a skin lesion of a patient with secondary syphilis following erythromycin treatment failure [11]–[13]. Previous studies [6], [14] have predicted that Nichols and SS14 strains represent sequentially distant groups of syphilis isolates.

Since the publication of the whole genome sequences of the Nichols and SS14 strains, these genome sequences have been shown to contain a significant number of sequencing errors [6], [7], [15]–[17]. Moreover, the majority of sequencing errors were similar in both Nichols and SS14 strains, which resulted from the use of the comparative genome sequencing approach (based on oligonucleotide probes) for determination of the SS14 genome sequence [9], [15]. In addition, sequencing errors in the Nichols and SS14 genomes complicated sequence comparisons with other, newly sequenced genomes [15], [16]. Since reliable, high quality whole genome sequences are required for precise genome annotation, we resequenced both the Nichols and SS14 genomes using the 454 and Illumina sequencing methods and reannotated both genomes. The corrected whole genome sequences resulted in considerable changes in treponemal proteomes and more clearly demonstrated that both the Nichols and SS14 strains represent different genetic subclusters of syphilis-causing treponemes.

## Materials and Methods

### Preparation of Chromosomal DNA

The chromosomal DNA used for genome sequencing in our study was the same stock used to sequence TPA SS14 strain originally [9]. For TPA strain Nichols, DNA was extracted from the same Nichols strain used originally for DNA extraction and genome sequencing [8]. Both DNA samples were provided by Steven J. Norris, The University of Texas Medical School in Houston, Houston, TX, USA. Whole genome amplification was achieved using the QIAGEN REPLI-g kit (QIAGEN, Valencia, CA, USA). The DNA concentrations used for sequencing were 598 ng/µl and 591 ng/µl for TPA strain Nichols and SS14, respectively.

### DNA Sequencing, Genome Annotation and Data Analysis

Both genomes were sequenced using the Illumina (Illumina GA, Illumina, San Diego, CA, USA) and 454 pyrosequencing (GS20, Roche, Basel, Switzerland) techniques. A total of 3 µg of amplified DNA was used for sequencing.

Sequencing reads were assembled using the CLC Bio Workbench (CLC Bio Katrinebjerg, Denmark). For the Nichols strain, 500 pyrosequencing contigs (ranging between 200 and 31,138 bp in length) and 345 Illumina contigs (200–26,924 bp) matching TPA Nichols reference genome sequence (AE000520.1) were obtained. Similarly, 199 pyrosequencing contigs (227–71,552 bp) and 201 Illumina contigs (201–43,429 bp) were assembled for the SS14 strain. Estimated sequencing coverage was 44 (454 pyrosequencing) and 55 (Illumina) for the TPA Nichols genome and 37 (454 pyrosequencing) and 68 (Illumina) for the TPA SS14 genome.

Sequentially related and repetitive regions of the treponemal genome were amplified separately using a GeneAmp XL PCR kit (Applied Biosystems, Foster City, CA, USA). For the *tprC* (TP_0117), *tprD* (TP_0131), *tprI* (TP_0620), *tprJ* (TP_0621), and *tprK* (TP_0897) regions, small insert libraries were prepared, as previously described [9], and Sanger sequenced. In addition, the following regions (defined previously as TPI amplicons [14], [18]), were Sanger sequenced directly from XL PCR products using a set of inner primers: TPI-11 (*tprC*), TPI-12 (*tprD*), TPI-13B (TP_0136), TPI-17A (rRNA operon 1), TPI-21B (rRNA operon 2), TPI-25A (*tprE*), TPI-25B-A (*tprF*), TPI-25B-B (*tprG*), TPI-26 (*tp92*), TPI-32B (*arp*), TPI-34 (TP_0470), TPI-38 (*mcp*), TPI-42A (TP_0548), TPI-48 (*tprI, tprJ*), and TPI-66A (*flaB*).

Drafts of the whole genome sequences were obtained by assembly of the pyrosequencing, Illumina and Sanger contigs with Nichols (AE000520.1) and SS14 GenBank sequences (CP000805.1) using the SeqMan program (DNASTAR, Madison, WI, USA). Any remaining discrepancies were Sanger sequenced following amplification with specific primers designed by Primer3 [19]. Complete genome sequences were annotated using the Geneious program [20]. All annotated genes were denoted by prefixes TPANIC_ and TPASS_2 for the Nichols and the SS14 genomes, respectively. The minimal gene length was set to 150 bp.

Both resequenced genomes were compared with reference Nichols (AE000520.1) and SS14 (CP000805.1) sequences and other treponemal genomes using Lasergene software package (DNASTAR, Madison, WI, USA) and Geneious. Identification of intrastrain heterogeneity in the SS14 and Nichols genomes was performed using BWA [21] and SAMtools [22]. Only genome sites with more than 20x coverage for each of the 454 and Illumina reads were tested for genetic heterogeneity. The frequency of the alternative allele was set to be 25% or higher for all reads. The nucleotide diversity was calculated using DnaSP software, version 5.10 [23]. Trees were built in Geneious using the Neighbor-Joining method (Tamura-Nei Genetic distance model).

### Nucleotide Accession Numbers

Resequenced whole genome sequences of TPA strains Nichols and SS14 were deposited in the GenBank database under accession numbers CP004010.2 and CP004011.1, respectively.

## Results

The genome sizes of resequenced (RS) TPA Nichols (Nichols-RS) and TPA SS14 (SS14-RS) strains were 1,139,633 bp and 1,139,569 bp, respectively. For TPA Nichols-RS and TPA SS14-RS genomes, 1,039 and 1,035 ORFs were predicted, respectively. At least one detectable sequencing error was found within 119 genes (11.5% of all genes in each genome).

### Error Identification and Correction

**Figure 1:**
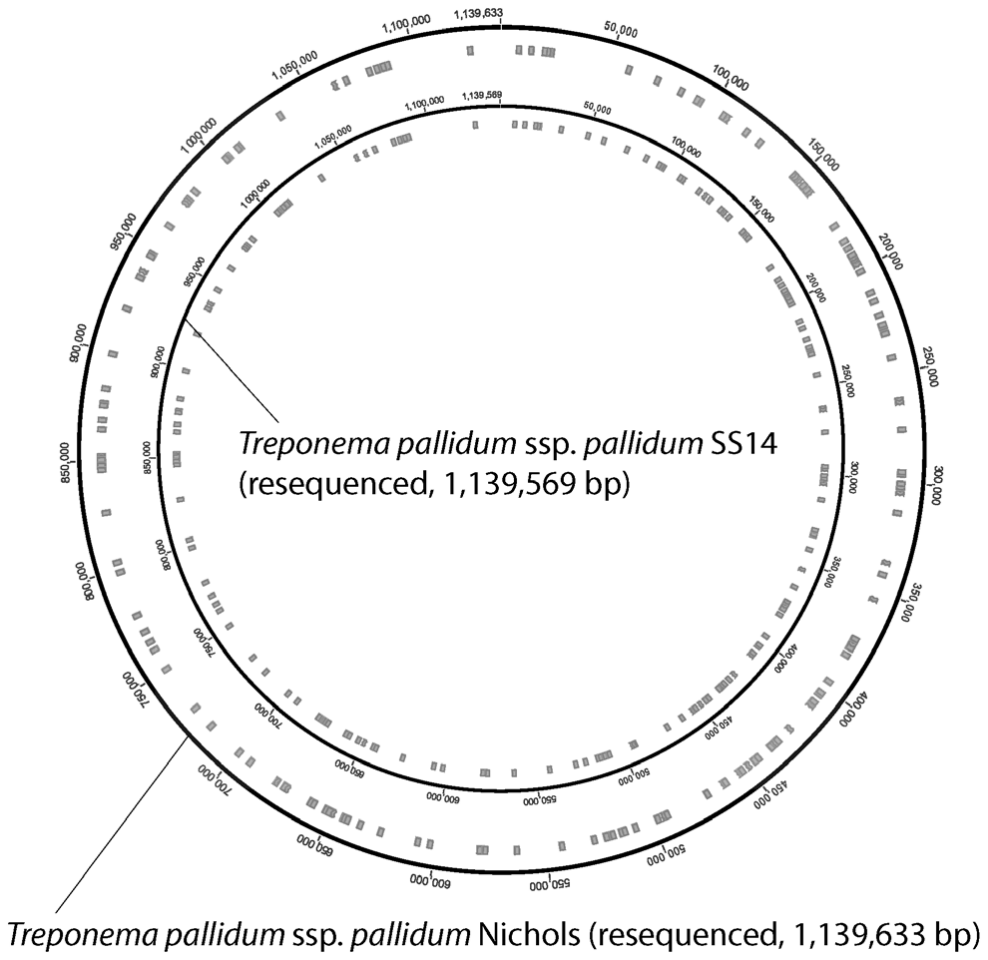
Distribution of identified errors in the original TPA Nichols and TPA SS14 genome sequences. Note that the chromosomal positions of identified errors are highly similar in both genomes as a result of the CGS sequencing approach used for sequencing the SS14 genome [9].

Sequencing errors were distributed evenly around the entire chromosome of the Nichols and SS14 genomes with slightly increased accumulation in the *tpr* genes and other known variable genomic loci [6] (Fig. 1).

**Figure 2:**
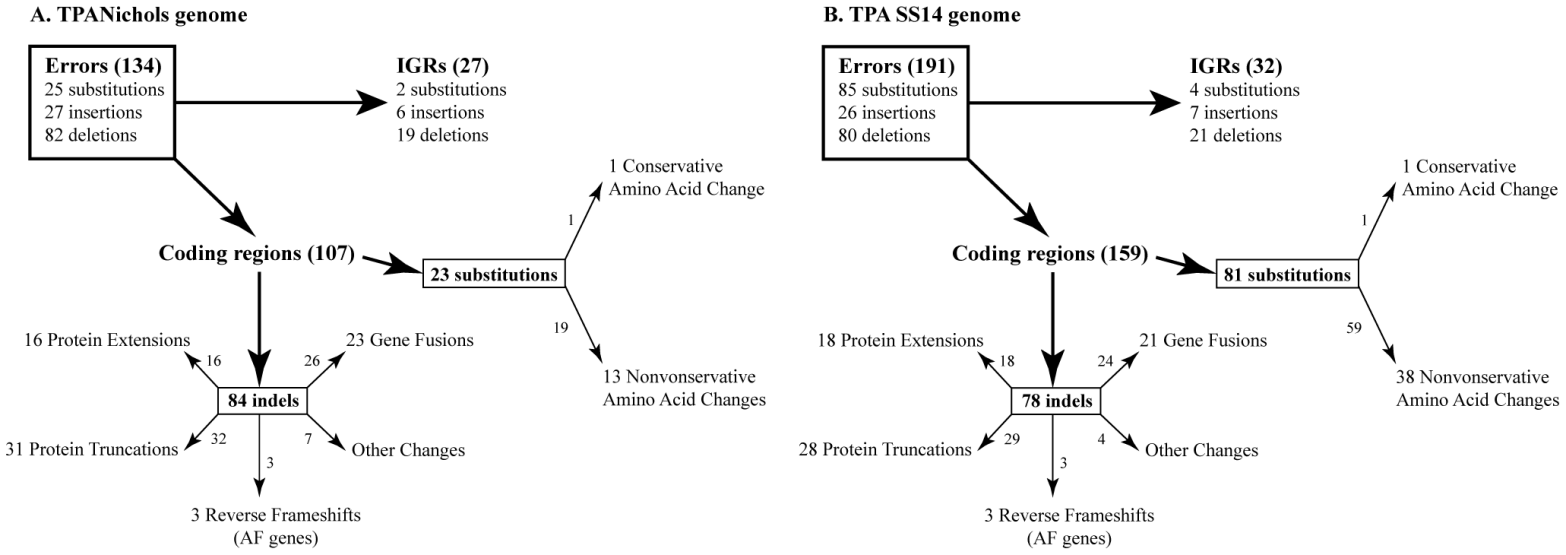
A schematic representation of errors identified in the original TPA Nichols and TPA SS14 genomes. The corresponding effects of error corrections are shown. AF stands for authentic frameshift. The group denoted “Other changes” includes in-frame errors; indels in the RNA region and indels in genes with annotated authentic frameshifts (see also Table S4). A. Effects of error corrections on the TPA Nichols genome. B. Effects of error corrections on the TPA SS14 genome. The numbers close to arrows indicate the number of nucleotide changes leading to changes in the proteome.

In the original TPA Nichols sequence (AE000520.1), a total of 134 errors were identified including 25 substitutions and 109 indels. One hundred and seven errors (79.9%) were found in the coding regions (23 substitutions and 84 indels) and 27 (20.1%) errors were located in the intergenic regions (IGRs) (Table S1). In the coding regions of the TPA Nichols-RS genome, correction of 19 substitution errors (out of 23; 82.6%) resulted in amino acid changes in the corresponding protein sequences including 13 non-conservative and 1 conservative change (Table S2). Correction of 84 indel errors (Fig. 2) in the coding regions led to truncations (31 proteins), extensions (16 proteins), fusions (23 proteins) (Table S3), reversion of frameshift mutations (3 proteins) and in frame indels (6 proteins). All of the gene fusions were previously reported [7], [15] with the exception of the gene fusion annotated as TPANIC_0007. All other sequenced TPA genomes (DAL-1, Chicago, Mexico A, SS14) showed fusion of three original genes, i.e. TP_0006, TP_0007 and TP_0008. In TPA Nichols genome substitution C→T (genome coordinate 7179) causes premature stop codon in TPANIC_0006, therefore fusion involves only the TP_0007 and TP_0008 loci. This position was found to be heterogeneous inside the Nichols strain and therefore both fusion variants exist. We identified T nucleotides in 108 Illumina reads (out of 153; 70.6%) and in 80 pyrosequencing reads (out of 127; 63%). In three cases, correction of indel errors (at the TP_0217, TP_0575 and TP_0866 loci) restored the reading frame and the originally annotated ORFs with authentic frameshifts were reannotated to genes. In all other treponemal genomes sequenced so far, these genes encode full length proteins without frameshifts [7], [15], [24], [25]. Finally, 6 indels resulted in other changes than reading frame mutations (i.e. insertion or deletion of amino acids) and 1 was found in the RNA region (Table S4). In addition to the above identified errors, the TPA Nichols-RS sequence was also improved in 57 positions, by identification of what had previously been ambiguous bases (Table S5).

In the TPA SS14-GenBank sequence (CP000805.1), a total of 191 errors were identified including 85 substitutions and 106 indels. One hundred and fifty nine (83.2%) errors were found in the coding regions (81 substitutions and 78 indels) while 32 (16.8%) errors were located in IGRs (Table S1). In the coding regions, correction of 59 substitution errors (out of 81; 72.8%) resulted in amino-acid changes in the corresponding protein including 38 non-conservative and 1 conservative change (Table S2). Correction of 24 (out of 78; 30.8%), 29 (37.1%) and 18 (23.1%) indel errors led to gene fusions, protein truncation and protein extension, respectively (Fig. 2). Similarly to the Nichols genome, frameshift mutations reverted to full length genes by correction of 3 indels in the TPASS_20217, TPASS_20575 and TPASS_20866 loci. In addition, 3 indels resulted in insertion or deletion of amino acids and 1 was found in RNA region (Table S4). Moreover, the TPA SS14 sequence was improved in 55 positions, by identification of previously ambiguous bases (Table S6). An overview of identified errors and the corresponding effects on the Nichols and SS14 proteomes is shown in Figure 2.

**Figure 3:**
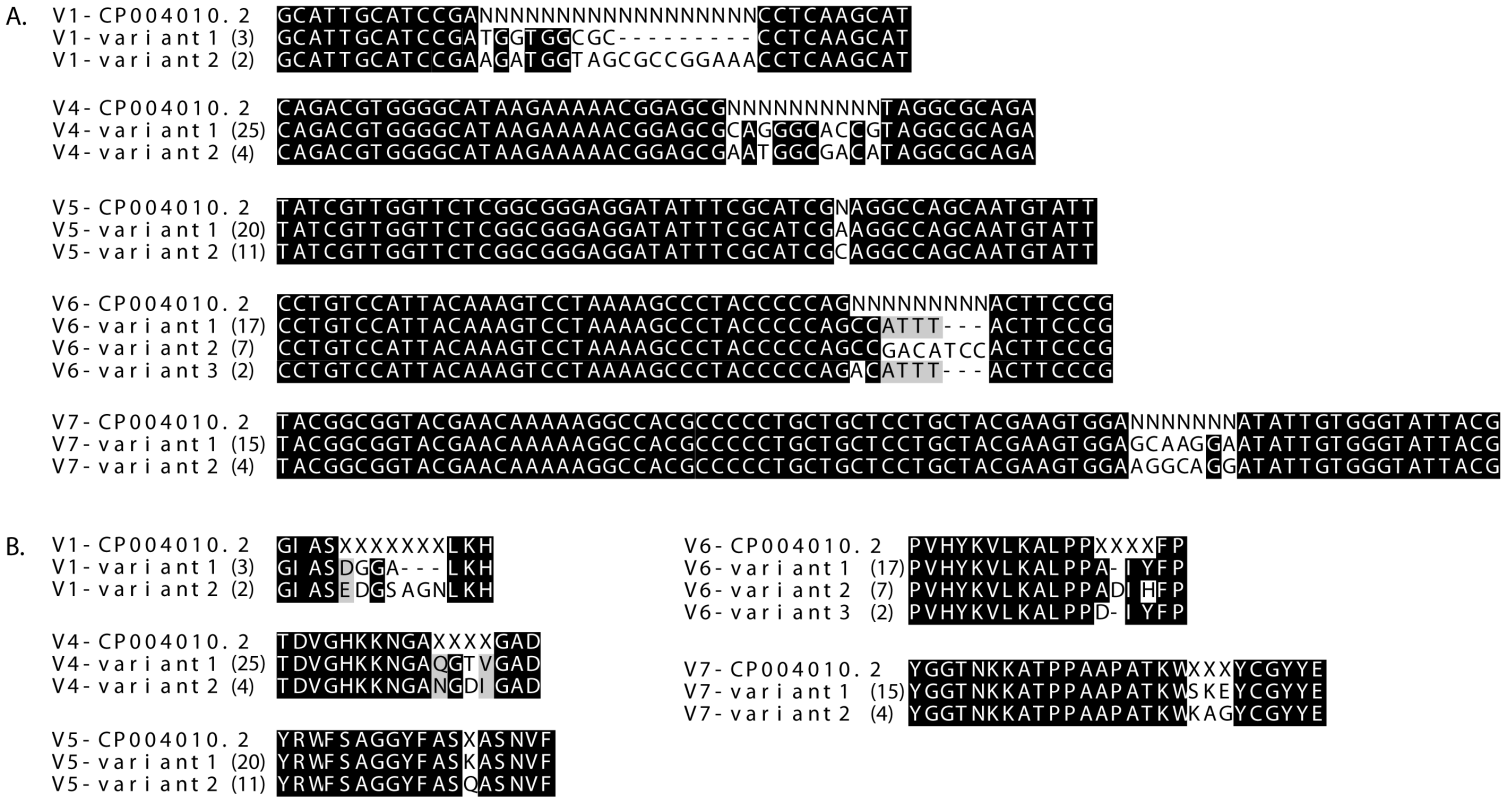
Nucleotide (amino acid) sequence alignments of TPA Nichols *tprK* (TprK) variable regions. Only sequence variants obtained by sequencing at least two independent clones are shown. Numbers in brackets correspond to number of clones with the same sequence variant. Because one could expect higher variability with more clones sequenced, Ns (Xs) were placed along the entire length of variable area. A) Nucleotide alignment of TPA Nichols *tprK* variable regions. B) Amino acid alignment of TPA Nichols TprK variable regions.

Sequencing of the small insert libraries prepared from DNA encoding the *tprK* gene revealed multiple sequence variants of *tprK,* not only in the SS14 strain, but also in the Nichols strain. Therefore, unlike the original Nichols sequence, variable nucleotides in V1, V4, V5, V6 and V7 *tprK* regions were denoted by the letter N to indicate detected variability in these positions in the Nichols-RS genome. Alignments of sequence variants identified in at least two independent clones are shown in Figure 3.

The corrected versions of the TPA SS14 and Nichols genomes differ in a number of ways. The point mutation in the 23S rRNA gene (A2058G), originally identified by Stamm and Bergen [26], was identified in the TPA SS14 but not in the Nichols genome. The TPASS_20326 (*tp92*) locus contains a sequence pattern typical for *Treponema pallidum* ssp. *endemicum* (TEN) strains (gene coordinates 1593–1649) [15], [27]. The TPASS_20868 gene encodes shortened FlaB protein (from 286 to 118 amino acid residues) due to a 7 bp deletion in this locus. The TP_0969 gene (encoding a putative outer membrane protein) is not annotated in the SS14-RS genome due to correction of an indel error which resulted in a shortened TPASS_0969 below the 150 bp threshold.

### Intrastrain Heterogeneity

Almost all previously described sites (n = 44) showing intrastrain heterogeneity in the SS14 genome [9] were confirmed, with the exception of two sites within the TPASS_20621 (*tprJ*) locus (gene coordinates 1794 and 1979) where only one version identical to the predominant sequence in the original SS14 sequence was found. In addition to these 42 verified sites in the SS14 genome, further intrastrain variable sites were found in the TPASS_20341 (*murC*), TPASS_20620 (*tprI*), TPASS_20621 (*tprJ*) and TPASS_20967 loci. All newly identified variable sites are presented in Table 1. Also, sequencing of small insert libraries and XL-PCR amplicons revealed intrastrain heterogeneity of the TPASS_20131 (*tprD*) locus, where both *tprD* and *tprD2* alleles [28] were found. While in the Nichols-RS genome, *tprC* and *tprD* alleles remain identical as previously reported [29], both *tprD* and *tprD2* alleles differ from the *tprC* sequence (9 nt and multiple sequence changes, respectively) in the SS14-RS genome.

**Table 1:** Newly identified genomic regions showing intrastrain heterogeneity in the TPA SS14-RS genome.

ORF (gene)	Sequence variants (amino acid variants)	Coordinates in the SS14-RS genome (CP004011.1)	Coordinates in the original SS14 genome (CP000805.1)
**TPASS_20131 (** ***tprD*** **)**	*tprD*/*tprD2* alleles	152541–153306	152528–153299
**TPASS_20341 (** ***murC*** **)**	C/T (P/L)	364888	364871
**TPASS_20620 (** ***tprI*** **)**	C/T (K/E)	674219	674191
	A/C (G/A)	674227	674199
	C/T (R/H)	674233	674205
**TPASS_20621 (** ***tprJ*** **)**	G/A (D/N)^a^	675157	675159
	C/T (D/N)^a^	675128	675130
**TPASS_20967**	variable number of “TCCTCCCCC” repeats: 2/3/4	1051840–1051866	1051729–1051755

Underlined variants were used in the improved whole genome sequence of the TPA SS14 strain (CP004011.1). Corresponding amino acid variants are shown in brackets.

a variant nucleotides are part of the same codon (therefore cause the same amino acid change).

In the Nichols genome, we confirmed the previously identified 1204-bp long insertion found in a subpopulation of the Nichols (Houston) strain [6], [14], [30]. In contrast, inspection of sites in the resequenced Nichols genome, corresponding to SS14-variable sites and as well as sites with previously ambiguously identified bases, revealed no variability in the Nichols-RS sequences.

### Comparison of Resequenced Genomes of TPA Strains Nichols and SS14 with other TPA Genomes

**Figure 4:**
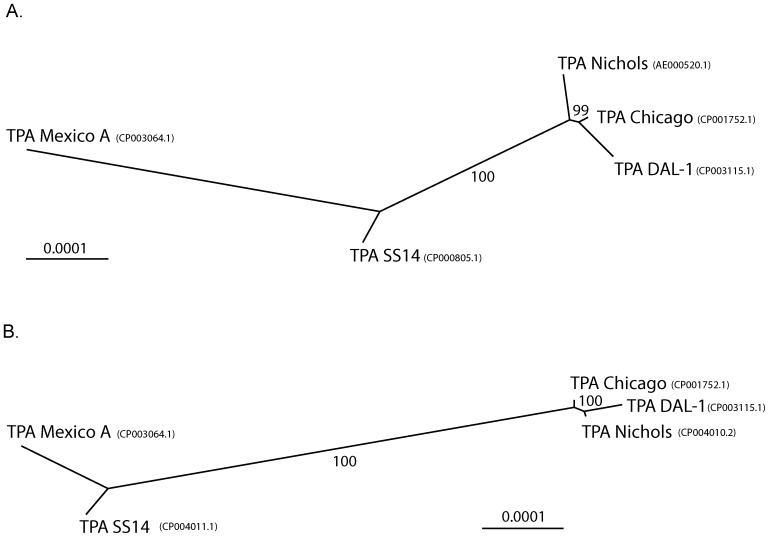
Unrooted trees constructed from whole genome sequences of TPA strains. Trees were constructed using the Neighbor-Joining method using the Tamura-Nei genetic distance model and 1,000 bootstrap replicates. The numbers above the branches show bootstrap support and the bar scale represents 0.0001 substitutions per target site. A. A tree constructed from the alignment of genomes from TPA strains Chicago (CP001752.1), DAL-1 (CP003115.1) and Mexico A (CP003064.1) with original versions of whole genome sequences of TPA Nichols (AE000520.1) and SS14 (CP000805.1). B. A tree constructed from alignment of genomes from TPA strains Chicago (CP001752.1), DAL-1 (CP003115.1) and Mexico A (CP003064.1) with the improved whole genome sequences of TPA Nichols-RS (CP004010.2) and SS14-RS (CP004011.1).

The whole genome sequences of Nichols and SS14 were compared to previously published genome sequences of TPA strains Mexico A (CP003064.1) [15], Chicago (CP001752.1) [24] and DAL-1 (CP003115.1) [25]. Because of high sequence diversities, *tprD* and *tprK* were excluded from analyses (Table 2 and 3). In contrast, the unrooted trees, shown in Fig. 4, were built from whole genome alignments (including the *tprD* and *tprK* gene sequences). Correction of sequencing errors resulted in a striking increase of genome relatedness within both the Nichols-like and SS14-like subclusters (Fig. 4), at the same time, both subclusters became more distantly related. Nucleotide diversity values also followed the same trend (Table 4).

**Table 2:** Effects of error correction on the improved TPA Nichols-RS genome with respect to comparisons with other TPA strains.

	The original Nichols genome (AE000520.1)			Nichols-RS genome (CP004010.2)			
	Sub	In	Del	Sub	In	Del	
**Chicago genome** (CP001752.1)	44	75	21	19 (43.2%)	8 (10.7%)	3 (14.3%)	
**DAL-1 genome** (CP003115.1)	105	91	35	26 (24.8%)	5 (5.5%)	5 (14.3%)	
**Mexico A genome** (CP003064.1)	438	94	38	363 (82.9%)	17 (18.1%)	16 (42.1%)	

The left part of the table shows former number of differences between the original TPA Nichols genome (AE000520.1) and other TPA genomes, the right part of the table shows verified number of differences between the improved TPA Nichols genome (CP004010.2) and other TPA genomes. Numbers in brackets show the percentage of verified differences between the improved TPA Nichols genome and other genomes compared to numbers observed during the comparison of the original Nichols genome and other TPA genomes. Because of high sequence diversities, *tprD* and *tprK* were excluded from the analyses.

sub, substitution; in, insertion; del, deletion.

**Table 3:** Effects of error correction on the improved TPA SS14-RS genome with respect to other TPA strains.

	The original SS14 genome (CP000805.1)			SS14-RS genome (CP004011.1)		
	Sub	In	Del	Sub	In	Del
**Chicago genome** (CP001752.1)	346	91	32	332 (96.0%)	17 (18.7%)	15 (46.9%)
**DAl-1 genome** (CP003115.1)	373	89	95	365 (97.9%)	18 (20.2%)	20 (21.1%)
**Mexico A genome** (CP003064.1)	175	85	28	91 (52.0%)	16 (18.8%)	11 (39.3%)
**The original Nichols genome** (AE000520.1)	327	14	18	–	–	–
**Nichols-RS genome** (CP004010.2)	–	–	–	350 (107.0%)	16 (114.3%)	17 (94.4%)

The left part of the table shows former number of differences between the original TPA SS14 genome (CP000805.1) and other TPA genomes, the right part of the table shows verified number of differences between the improved TPA SS14 genome (CP004011.1) and other TPA genomes. Numbers in brackets show the percentage of verified differences between the improved TPA SS14 genome and other genomes compared to numbers observed during the comparison of the original SS14 genome and other TPA genomes. Because of high sequence diversities, *tprD* and *tprK* were excluded from the analyses. Please note that the number of sequence differences between TPA SS14 and Nichols genomes increased after error correction.

sub, substitution; in, insertion; del, deletion.

**Table 4:** Calculated nucleotide diversities (π ± standard deviation) between individual TPA strains and the original and resequenced versions of the Nichols and SS14 strains.

	The original Nichols genome(AE000520.1)	Nichols-RS genome(CP004010.2)	The original SS14 genome(CP000805.1)	SS14-RS genome(CP004011.1)
**Chicago genome** (CP001752.1)	0.00004±0.00002	0.00003±0.00002	0.00031±0.00016	0.00059±0.00030
**DAL-1 genome** (CP003115.1)	0.00009±0.00005	0.00005±0.00002	0.00035±0.00017	0.00064±0.00032
**Mexico A genome** (CP003064.1)	0.00070±0.00035	0.00066±0.00033	0.00045±0.00023	0.00015±0.00007
**Nichols-RS genome** (CP004010.2)	–	–	0.00031±0.00016	0.00060±0.00030
**SS14-RS genome** (CP004011.1)	0.00063±0.00032	0.00060±0.00030	–	–
**The original Nichols genome**(AE000520.1)	–	–	0.00031±0.00016	0.00063±0.00032

## Discussion

In the 1998, the TPA Nichols strain was one of the first completed bacterial genomes with an average coverage of Sanger reads of 6.7x [8] and an estimated average error rate close to 10^−4^. Based on this estimation, one can expect over 100 sequencing errors scattered throughout the genome. Strain SS14 was completely sequenced in 2008, ten years after the Nichols genome. Since sequencing of the TPA SS14 genome was performed using the CGS approach [9], errors present in the Nichols genome were replicated to the whole genome sequence of the SS14 strain. Sequencing errors in both Nichols and SS14 genomes were distributed evenly around the entire chromosome with minor accumulations in the *tpr* genes and other known variable genomic loci [6] (Fig. 1). It is likely that this minor accumulation of sequencing errors is a result of difficulties encountered during Sanger sequencing of the paralogous regions of the Nichols genome. Accumulation of whole genome sequencing results in recent years [6] has revealed that treponemes are highly clonal organisms and subtle differences in their genome are responsible for profound differences in the clinical manifestations of diseases and in the host range [7]. Therefore, high quality genomic sequences are needed to analyze genome differences between TPA and TPE strains. Moreover, the presence of sequencing errors has complicated comparisons of TPA Nichols and SS14 strains with other recently completed treponemal genomes. Therefore we resequenced both Nichols and SS14 genomes with a combined average coverage of more than 90x with subsequent analysis of primary sequencing reads for all discrepant nucleotides. In other re-sequencing projects, analysis of Illumina reads revealed that at a coverage of 20x, the average number of errors per kbp is close to zero and the average number of errors does not decrease any further [31]. We therefore estimate that the number of remaining sequencing errors in the Nichols-RS and SS14-RS genomes is between 0 and a few nucleotides per genome.

A total of 134 errors in TPA strain Nichols and 191 errors in TPA strain SS14 reference genomes were found (affecting 119 annotated genes; 11.5%), which is close to a previous estimation [6]. In the Nichols genome, sequencing errors were previously reported: 1 [17], 32 [6], 36 [7], 64 [16], and 93 [15] and confirmed in the present study. With the exception of a slightly increased accumulation of sequencing errors in the *tpr* genes and other known variable genomic loci [6], sequencing errors were distributed evenly across the entire treponemal chromosome (Fig. 1). These data suggest that most of the identified sequencing errors occurred by chance with no detectable bias in chromosome localization.

Compared to substitutions, the detected predominance of indel errors resulted in more profound changes in the predicted treponemal proteome. Resequencing of TPA Nichols and SS14 strains revealed considerable changes in annotations of 65 and 63 proteins, respectively, including elongations, truncations and fusions.

Unlike in the original Nichols genome, we found variability in V1, V4, V5, V6 and V7 variable regions of *tprK* gene. A similar degree of *tprK* variability was also observed in the Nichols strain UNC [32] and the authors suggested that the observed variability is a result of routinely administered hydrocortisone acetate used for downregulation of the patient’s immune response. TprK variants that arise during infection were maintained [32]. In contrast, *tprK* variable regions in the Nichols (Seattle strain) were reported to remain conserved [33]. Nevertheless, *tprK* variability found in the Nichols genome was considerably lower compared to the variability detected in the *tprK* of the SS14 strain.

Important differences between TPA SS14 and Nichols genomes exist in several loci including 23S rRNA, TP_0326 (*tp92*), TP_0868 (*flaB*) and TP_0127. In strain SS14 the presence of the A2058G point mutation, in 23S rRNA gene, is responsible for its resistance to macrolides [26]. The TPASS_20326 locus contains a sequence pattern typical for *Treponema pallidum* ssp. *endemicum* (TEN) strain [15], [27]. TPASS_20326 encodes a BamA orthologue involved in membrane biogenesis [34]. The “TEN-like” sequence pattern is located in the outer membrane spanning β-barrel domain [34] and suggests recombination between TPA and TEN strains [15]. Until now, the SS14 is the only strain with a shortened version of one of the FlaB proteins. A frameshift in the TPANIC_0127 locus resulted in a change in primary structure of the protein similar to the TPA0127.3 variant described by Giacani *et al.*, which encodes MutS1 homologue [35].

In addition to the above mentioned differences, dozens of genomic sites showing intrastrain heterogeneity were identified in the SS14 genome. The occurrence of multiple alleles in a population of a bacterial pathogen suggests the presence of contingency genes and an antigenic variation [36]. To filter sequencing errors, identification of intrastrain heterogeneity in the Nichols and SS14 genomes was limited to sites with more than 20x coverage for each of the 454 and Illumina reads and to a 25%, or higher, frequency of alternative alleles. It is therefore possible that additional sequencing and further genome sequence analyses will reveal additional heterogenic regions in the SS14 strain. In general, the presence of different sequences at the same locus among bacteria of the same strain (e.g. the presence of both *tprD* and *tprD2* alleles known to encode for outer membrane proteins [37]) could represent an important mechanism for avoiding host immune responses. The presence of alternative *tprD* alleles could be a result of gene conversion event between *tpr* loci of the same strain [5] or by a recombination event between TP strains as was suggested for TP_0326 an TP_0488 loci of the Mexico A strain [15]. A recent study by Centurion-Lara *et al.* [13] did not support our findings about two *tprD* alleles in the TPA SS14 strain. This could be explained by higher numbers of sequenced clones in our study which lead to the identification of *tprD*. We obtained sequencing data for 51 clones, with 32 sequences corresponding to *tprD* and 19 to *tprD2*. Centurion-Lara *et al.* sequenced less clones (2 to 10 clones for each TP strain) and so the smaller sample size may have been the reason for identifying only one allele of *tprD* gene.

Interestingly, intrastrain heterogeneity identified in the Nichols genome was significantly lower compared to the SS14 strain. Although the reason for this is unknown, one possible explanation involves the number of years that elapsed between the isolation of the strains from patients. However, other reasons including inherent differences in genome structure and regulation cannot be excluded.

It is known that treponemes detected in clinical material either genetically belong to Nichols-like or SS14-like strains [6], [14]. While clinical isolates genetically related to both Nichols and SS14 strains have been detected in the USA [38], Ireland [38], China [39] and Taiwan [40], only Nichols-like isolates were detected among 20 typeable samples from Madagascar [38], and only SS14-like strain variants were found among 64 patients with typeable treponemal DNA from Central Europe [41] and among 5 patients typed in London [42]. Although the reason for different geographical occurrences of Nichols-like or SS14-like strains is unknown, epidemiologic reasons as well as existing genome differences between Nichols and SS14 strains should be considered.

Correction of 134 and 191 errors in the Nichols and SS14 genome, respectively, resulted in an increased separation of both syphilis treponeme subclusters (Fig. 4). As it turns out, genetic diversity within subgroups of syphilis strains is less than originally reported and that both subgroups of Nichols-like and SS14-like strains are more distinct at the same time. Nucleotide diversity between TPA Nichols and SS14 subgroups increased after error correction by almost two fold; from 0.00031±0.000016 to 0.00060±0.000030.

Several bacterial genomes recently resequenced by next generation sequencing methods [43]–[45] revealed numerous errors in the previously published sequences. Since these errors could significantly affect protein annotations and complicate further comparative studies, revisions of genome sequences are of crucial importance. Taken together, resequencing of TPA Nichols and SS14 genomes considerably improved genome annotations and resulted in correction of more than 11% of the corresponding proteome annotations. Resequencing of the genomes more clearly revealed the distinctness of the two separate genetic subclusters of syphilis-causing treponemes, despite the fact that treponemes from both subgroups cause the same disease in humans.

## Supporting Information

Table S1 **List of errors identified in the intergenic regions (IGRs) in the original TPA Nichols and TPA SS14 genomes.**
(DOCX)

Table S2 **List of substitution errors identified in the original TPA Nichols and TPA SS14 genomes.**
(DOCX)

Table S3 **Gene fusions identified in the resequenced versions of the TPA Nichols and SS14 genomes as a result of in-del corrections.**
(DOCX)

Table S4 **List of indel errors identified in the original TPA Nichols and SS14 genomes.**
(DOCX)

Table S5 **List of all errors identified in the coding regions of the original TPA Nichols sequence (AE000520.1).**
(XLSX)

Table S6 **List of all errors identified in the intergenic regions of the original TPA SS14 sequence (CP000805.1).**
(XLSX)
