# Supplementary material for: Resequencing of Treponema pallidum ssp. pallidum Strains Nichols and SS14: Correction of Sequencing Errors Resulted in Increased Separation of Syphilis Treponeme Subclusters
Source: PLoS One. 2013 Sep 10;8(9):e74319. doi: 10.1371/journal.pone.0074319 (PMC3769245; doi:10.1371/journal.pone.0074319)
Supplement: Table S1 — List of errors identified in the intergenic regions (IGRs) in the original TPA Nichols and TPA SS14 genomes. (DOCX) [file pone.0074319.s001.docx]

Table S1. List of errors identified in the intergenic regions (IGRs) in the original TPA Nichols and TPA SS14 genomes

| **Affected IGR in the original Nichols sequence** | **Affected IGR in the original SS14 sequence** | **Coordinates in the original Nichols sequence (AE000520.1)** | **Coordinates in Nichols-RS sequence (CP004010.2)** | **Coordinates in the original SS14 sequence (CP000805.1)** | **Coordinates in the SS14-RS sequence (CP004011.1)** | **Error type in the Nichols/SS14 original sequence** | **Resulting change in the Nichols/SS14-RS sequence** |
| --- | --- | --- | --- | --- | --- | --- | --- |
| - | TPASS_0026 - 0027 | - | - | 34076 - 34077 | 34083 | del (C) | in (C) |
| TP_0082 - 0083 | TPASS_0082 - 0083 | 93127 - 93128 | 93138 | 93124 - 93125 | 93137 | del (C) | in (C) |
| - | TPASS_0084 - 0085 | - | - | 94715 - 94716 | 94730 | del (C) | in (C) |
| - | TPASS_0107 - 0108 | - | - | 122225 | 122238 - 122239 | in (C) | del (C) |
| - | TPASS_0117 - 0118 | - | - | 136732 - 136733 | 136744 - 136745 | in (CC) | del (CC) |
| - | TPASS_0121 - 0122 | - | - | 140946 - 140947 | 140958 - 140959 | del (CC) | in (CC) |
| TP_0162 - 0163 | TPASS_0162 - 0163 | 184504 - 184505 | 185656 | 185687 - 185688 | 185696 | del (C) | in (C) |
| TP_0222 - 0223 | TPASS_0222 - 0223 | 227443 - 227444 | 228597 | 228626 - 228627 | 228639 | del (C) | in (C) |
| TP_0222 - 0223 | TPASS_0222 - 0223 | 227443 | 228598 | 228627 | 228640 | sub (G→A) | sub (A→G) |
| TP_0233 - t013 | TPASS_0233 - t0013 | 241857 - 241858 | 243013 | 243040 - 243041 | 243055 | del (C) | in (C) |
| TP_0277 - 0278 | TPASS_0277 - 0278 | 294000 - 294001 | 295157 | 295183 - 295184 | 295199 | del (C) | in (C) |
| - | TPASS_0316 - 0317 | - | - | 333542 | 333559 | sub (T→C) | sub (C→T) |
| - | TPASS_0316 - 0317 | - | - | 333572 | 333589 | sub (G→A) | sub (A→G) |
| TP_0349 - 0350 | TPASS_0349 - 0350 | 374555 | 375713 - 375714 | 375735 | 375749 - 375750 | in (C) | del (C) |
| TP_0380 - 0381 | TPASS_0380 - 0381 | 407166 - 407167 | 408327 | 408346 - 408347 | 408363 | del (G) | in (G) |
| TP_0380 - 0381 | TPASS_0380 - 0381 | 407206 - 407207 | 408368 | 408386 - 408387 | 408404 | del (G) | in (G) |
| TP_0381 - 0382 | - | 407943 | 409104 - 409105 | - | - | in (C) | del (C) |
| - | TPASS_0381 - 0382 | - | - | 409132 - 409135 | 409149 - 409150 | in (CCCC) | del (CCCC) |
| TP_0396 - 0397 | TPASS_0396 - 0397 | 422318 | 423478 - 423479 | 423498 | 423511 - 423512 | in (G) | del (G) |
| TP_0408 - 0409 | TPASS_0408 - 0409 | 434977 - 434978 | 436217 - 436218 | 436157 - 436158 | 436170 - 436171 | del (GC) | in (GC) |
| TP_0423 - 0424 | TPASS_0432 - 0424 | 451900 - 451901 | 453062 | 453080 - 453081 | 453095 | del (G) | in (G) |
| TP_0461 - 0462 | TPASS_0461 - 0462 | 491019 - 491020 | 492601 | 492618 - 492619 | 492633 | del (G) | in (G) |
| TP_0485 - 0486 | - | 517500 - 517501 | 519085 | - | - | del (C) | in (C) |
| TP_0535 - 0536 | TPASS_0535 - 0536 | 578366 - 578367 | 579955 | 579798 - 579799 | 579820 | del (C) | in (C) |
| TP_0598 - 0599 | TPASS_0598 - 0599 | 650905 - 650906 | 652503 | 652359 - 652360 | 652386 | del (G) | in (G) |
| TP_t30 - 0608 | TPASS_t0030 - 0608 | 658396 - 658397 | 659995 | 659850 - 659851 | 659878 | del (C) | in (C) |
| TP_0621 - 0622 | - | 675241 | 676838 - 676839 | - | - | in (C) | del (C) |
| TP_0651 - 0652 | TPASS_0651 - 0652 | 715538 - 715539 | 717137 | 716992 - 716993 | 717023 | del (C) | in (C) |
| TP_0683 - 0684 | TPASS_0683 - 0684 | 748249 | 759848 | 749697 | 749728 | sub (A→T) | sub (T→A) |
| TP_0781 - 0782 | TPASS_0781 - 0782 | 848867 - 848868 | 850470 | 850315 - 850316 | 850350 | del (G) | in (G) |
| TP_0865 - 0866 | - | 944866 - 944867 | 946471 | - | - | del (C) | in (C) |
| TP_0893 - 0894 | TPASS_0893 - 0894 | 972616 | 974221 - 974222 | 974061 | 974197 - 974198 | in (G) | del (G) |
| - | TPASS_0924 - t0042 | - | - | 1006824 - 1006825 | 1006931 | del (C) | in (C) |
| TP_0926 - 0927 | TPASS_0926 - 0927 | 1007875 - 1007876 | 1009481 | 1009320 - 1009321 | 1009428 | del (G) | in (G) |
| TP_0949 - 0950 | TPASS_0949 - 0950 | 1032143 - 1032144 | 1033761 | 1033588 - 1033589 | 1033698 | del (G) | in (G) |
| TP_0975 - 0976 | TPASS_0975 - 0976 | 1059221 | 1060839 - 1060840 | 1060666 | 1060775 - 1060776 | in (G) | del (G) |

sub, substitution; in, insertion; del, deletion
