# Supplementary material for: Resequencing of Treponema pallidum ssp. pallidum Strains Nichols and SS14: Correction of Sequencing Errors Resulted in Increased Separation of Syphilis Treponeme Subclusters
Source: PLoS One. 2013 Sep 10;8(9):e74319. doi: 10.1371/journal.pone.0074319 (PMC3769245; doi:10.1371/journal.pone.0074319)
Supplement: Table S2 — List of substitution errors identified in the original TPA Nichols and TPA SS14 genomes. (DOCX) [file pone.0074319.s002.docx]

Table S2. List of substitution errors identified in the original TPA Nichols and TPA SS14 genomes

| **Affected region in the original Nichols sequence (coding strand)** | **Affected region in the original SS14 sequence (coding strand)** | **Coordinates in the original Nichols sequence (AE000520.1)** | **Coordinates in Nichols-RS sequence (CP004010.2)** | **Coordinates in the original SS14 sequence (CP000805.1)** | **Coordinates in the SS14-RS sequence (CP004011.1)** | **Substitution error in the original Nichols/SS14- sequence** | **Resulting change in the Nichols/SS14-RS sequence** | **Resulting amino acid change in the Nichols/SS14-RS protein sequence** |
| --- | --- | --- | --- | --- | --- | --- | --- | --- |
| TP_0020(+) | **-** | 22506 | 22512 | - | - | G→C | C→G | T→S |
| TP_0049(-) | TPASS_0049(-) | 58312 | 58318 | 58309 | 58318 | G→A | A→G | none |
| TP_0076(+) | - | 83983 | 83991 | - | - | G→C | C→G | L→V |
| TP_0106(+) | TPASS_0106(-) | 119199 | 119210 | 119196 | 119210 | G→C | C→G | V→L |
| TP_0106(+) | TPASS_0106(-) | 119200 | 119211 | 119197 | 119211 | C→G | G→C | F→L |
| - | TPASS_0117(-) | - | - | 135217 | 135230 | A→G | G→A | T→I |
| - | TPASS_0117(-) | - | - | 135224 | 135237 | A→G | G→A | P→S |
| - | TPASS_0117(-) | - | - | 135232 | 135245 | A→G | G→A | A→V |
| - | TPASS_0117(-) | - | - | 135236 | 135249 | T→C | C→T | G→R |
| TP_0154(+) | TPASS_0154(+) | 176236 | 177387 | 177419 | 177427 | G→C | C→G | R→A |
| TP_0154(+) | TPASS_0154(+) | 176237 | 177388 | 177420 | 177428 | C→G | G→C | V→L*^a^* |
| TP_0154(+) | TPASS_0154(+) | 176238 | 177389 | 177421 | 177429 | G→C | C→G | V→L*^a^* |
| TP_0154(+) | TPASS_0154(+) | 176239 | 177390 | 177422 | 177430 | C→G | G→C | V→L*^a^* |
| - | TPASS_0313(+) | - | - | 330887 | 330904 | G→T | T→G | F→V |
| - | TPASS_0314(-) | - | - | 331525 | 331542 | C→G | G→C | none |
| - | TPASS_0314(-) | - | - | 331533 | 331550 | C→A | A→C | F→V |
| - | TPASS_0317(-) | - | - | 333716-334139 | 333733-334156 | ME | MSC | MSC |
| TP_0350(+) | TPASS_0350(+) | 375095 | 376253 | 376275 | 376289 | G→C | C→G | P→A |
| TP_0379(-) | TPASS_0379(-) | 402741 | 403901 | 403921 | 403937 | G→A | A→G | I→S |
| TP_0379(-) | TPASS_0379(-) | 402742 | 403902 | 403922 | 403938 | A→T | T→A | none |
| - | TPASS_0402(+) | - | - | 428615 | 428628 | T→C | C→T | P→L |
| TP_0468(-) | - | 495739 | 497320 | - | - | G→A | A→G | M→A |
| TP_0469(-) | - | 495739 | 497320 | - | - | G→A | A→G | M→A |
| TP_0468(-) | - | 495740 | 497321 | - | - | C→T | T→C | none |
| TP_0469(-) | - | 495740 | 497321 | - | - | C→T | T→C | none |
| TP_0469(-) | - | 495741 | 497322 | - | - | A→G | G→A | none |
| TP_0583(-) | - | 633825 | 635418 | - | - | T→G | G→T | none*^b^* |
| TP_0591(-) | TPASS_0591(-) | 641838 | 643432 | 643288 | 643315 | G→A | A→G | V→F |
| TP_0591(-) | TPASS_0591(-) | 641840 | 643434 | 643290 | 643317 | A→C | C→A | F→L*^a^* |
| TP_0591(-) | TPASS_0591(-) | 641841 | 643435 | 643291 | 643318 | C→G | G→C | F→L*^a^* |
| - | TPASS_0619(-) | - | - | 672099-672180 | 672127-672208 | ME | MSC | MSC |
| TP_0633(+) | TPASS_0633(+) | 691588 | 693185 | 693042 | 693071 | G→T | T→G | Y→D |
| TP_0805(-) | TPASS_0805(-) | 874773 | 876375 | 876221 | 876255 | G→C | C→G | R→A*^a^* |
| TP_0805(-) | TPASS_0805(-) | 874774 | 876376 | 876222 | 876256 | C→G | G→C | R→A*^a^* |
| TP_0983(+) | TPASS_0983(+) | 1066104 | 1067722 | 1067549 | 1067658 | G→C | C→G | P→R |
| - | TPASS_1030(-) | - | - | 1125566 | 1125677 | G→C | C→G | none |
| - | TPASS_1030(-) | - | - | 1125689 | 1125801 | T→G | G→T | H→R |
| - | TPASS_1030(-) | - | - | 1125690 | 1125802 | C→T | T→C | none |
| - | TPASS_1030(-) | - | - | 1125694 | 1125806 | G→A | A→G | none |

ME, multiple errors; MSC, major sequence changes; *^a^*Substitution is causing amino acid change together with adjacent substitutions; *^b^*ORF is not annotated in the Nichols/SS14-RS genomes.
