# Supplementary material for: Resequencing of Treponema pallidum ssp. pallidum Strains Nichols and SS14: Correction of Sequencing Errors Resulted in Increased Separation of Syphilis Treponeme Subclusters
Source: PLoS One. 2013 Sep 10;8(9):e74319. doi: 10.1371/journal.pone.0074319 (PMC3769245; doi:10.1371/journal.pone.0074319)
Supplement: Table S3 — Gene fusions identified in the resequenced versions of the TPA Nichols and SS14 genomes as a result of in-del corrections. (DOCX) [file pone.0074319.s003.docx]

**Table S3. Gene fusions identified in the resequenced versions of the TPA Nichols and SS14 genomes as a result of indel corrections**

| **Fused genes in the original annotation of the Nichols strain (AE000520.1)** | **ORF annotation in the Nichols-RS genome (CP004010.2)** | **Fused genes in the original annotation of the SS14 strain (CP000805.1)** | **ORF annotation in the SS14-RS genome (CP004011.1)** |
| --- | --- | --- | --- |
| N/A* | N/A | TPASS_0006 - TPASS_0008 | TPASS_0006 |
| TP_0007, TP_0008 | TPANIC_0007 | N/A | N/A |
| TP_0013, TP_0014 | TPANIC_0013 | TPASS_0013, TPASS_0014 | TPASS_0013 |
| TP_0018, TP_0019 | TPANIC_0018 | TPASS_0018, TPASS_0019 | TPASS_0018 |
| TP_0172, TP_0173 | TPANIC_0172 | TPASS_0172, TPASS_0173 | TPASS_0172 |
| TP_0174, TP_0175, TP_0176 | TPANIC_0174 | TPASS_0174, TPASS_0175, TPASS_0176 | TPASS_0174 |
| N/A | N/A | TPASS_0179, TPASS_0180 | TPASS_0179 |
| TP_0284, TP_0285 | TPANIC_0284 | TPASS_0284, TPASS_0285 | TPASS_0284 |
| TP_0286, TP_0287 | TPANIC_0286 | TPASS_0286, TPASS_0287 | TPASS_0286 |
| TP_0288, TP_0289 | TPANIC_0288 | TPASS_0288, TPASS_0289 | TPASS_0288 |
| TP_0299, TP_0300 | TPANIC_0300 | TPASS_0299, TPASS_0300 | TPASS_0300 |
| TP_0324, TP_0325 | TPANIC_0324 | TPASS_0324, TPASS_0325 | TPASS_0324 |
| TP_0377, TP_0378 | TPANIC_0377 | TPASS_0377, TPASS_0378 | TPASS_0377 |
| TP_0419, TP_0420 | TPANIC_0419 | TPASS_0419, TPASS_0420 | TPASS_0419 |
| TP_0433, TP_0434 | TPANIC_0433 | TPASS_0433** | TPASS_0433 |
| TP_0462, TP_0463 | TPANIC_0462 | TPASS_0462, TPASS_0463 | TPASS_0462 |
| TP_0468, TP_0469 | TPANIC_0468 | TPASS_0468** | TPASS_0468 |
| TP_0481, TP_0482 | TPANIC_0481 | TPASS_0481, TPASS_0482 | TPASS_0481 |
| TP_0587, TP_0588 | TPANIC_0587 | TPASS_0587, TPASS_0588 | TPASS_0587 |
| TP_0597, TP_0598 | TPANIC_0598 | TPASS_0598** | TPASS_0598 |
| TP_0702, TP_0703 | TPANIC_0702 | TPASS_0702, TPASS_0703 | TPASS_0702 |
| TP_0781, TP_0782 | TPANIC_0781 | TPASS_0781, TPASS_0782 | TPASS_0781 |
| TP_0859, TP_0860 | TPANIC_0859 | TPASS_0859, TPASS_0860 | TPASS_0859 |
| TP_0899, TP_0900 | TPANIC_0899 | TPASS_0899, TPASS_0900 | TPASS_0899 |
| TP_0928, TP_0929 | TPANIC_0928 | TPASS_0928, TPASS_0929 | TPASS_0928 |

*N/A, not applicable; **the fusion (corresponding to fusion of the originally annotated Nichols orthologs) was already annotated in the published SS14 genome (Matějková et al. 2008).
