# Supplementary material for: Resequencing of Treponema pallidum ssp. pallidum Strains Nichols and SS14: Correction of Sequencing Errors Resulted in Increased Separation of Syphilis Treponeme Subclusters
Source: PLoS One. 2013 Sep 10;8(9):e74319. doi: 10.1371/journal.pone.0074319 (PMC3769245; doi:10.1371/journal.pone.0074319)
Supplement: Table S4 — List of indel errors identified in the original TPA Nichols and SS14 genomes. (DOCX) [file pone.0074319.s004.docx]

Table S4. List of indel errors identified in the original TPA Nichols and TPA SS14 genomes

| **Gene affected in the original Nichols sequence (coding strand)** | **Gene affected in the original SS14 sequence (coding strand)** | **Coordinates in the original Nichols sequence (AE000520.1)** | **Coordinates in Nichols-RS sequence (CP004010.2)** | **Coordinates in the original SS14 sequence (CP000805.1)** | **Coordinates in the SS14-RS sequence (CP004011.1)** | **Error type in the Nichols/SS14 original sequence** | **Resulting change in the Nichols/SS14-RS sequence** | **Resulting amino acid change in the Nichols-RS and SS14-RS protein sequence** |
| --- | --- | --- | --- | --- | --- | --- | --- | --- |
| - | TPASS_0006(+) | - | - | 7939 - 7940 | 7940 | del (C) | in (C) | GF |
| TP_0007(+) | - | 7938 - 7939 | 7940 | - | - | del (C) | in (C) | GF |
| TP_0013(+) | TPASS_0013(+) | 13195 - 13196 | 13199 - 131200 | 13194 - 13195 | 13196 - 13197 | del (GG) | in (GG) | GF |
| TP_0014(+) | TPASS_0014(+) | 13196 - 13196 | 13200 - 131200 | 13194 - 13195 | 13196 - 13197 | del (GG) | in (GG) | GF*^a^* |
| TP_0018(+) | TPASS_0018(+) | 19431 - 19432 | 19436 | 19429 - 19430 | 19433 | del (G) | in (G) | EP*^b^* |
| TP_0018(+) | TPASS_0018(+) | 21231 - 21232 | 21237 | 21229 - 21230 | 21234 | del (G) | in (G) | GF |
| TP_0018(+) | TPASS_0018(+) | 21360 - 21361 | 21367 | 21358 - 21359 | 21364 | del (A) | in (A) | GF |
| - | TPASS_0040(+) | - | - | 49361 - 49362 | 49369 | del (G) | in (G) | EP |
| TP_0049(-) | TPASS_0049(-) | 58311 - 58312 | 58319 | 58308 - 58309 | 58317 | del (C) | in (C) | TP |
| TP_0065(+) | TPASS_0065(+) | 72281 - 72282 | 72290 | 72279 - 72280 | 72289 | del (G) | in (G) | TP |
| TP_0076() | TPASS_0076(+) | 84205 - 84206 | 84214 | 84202 - 84203 | 84213 | del (G) | in (G) | EP |
| TP_0082(-) | TPASS_0082(-) | 92170 - 92171 | 92181 | 92168 - 92169 | 92180 | del (G) | in (G) | TP |
| TP_0083(+) | TPASS_0083(+) | 94140 - 94141 | 94152 | 94137 - 94138 | 94151 | del (C) | in (C) | TP |
| TP_0098(-) | TPASS_0098(-) | 107082 | 107093 - 107094 | 107079 | 107093 - 107094 | in (G) | del (G) | EP |
| TP_0126(-) | - | 148340 | 148350 - 148351 | - | - | in (C) | del (C) | TP |
| TP_0127(+) | - | 148945 | 150158 - 150159 | - | - | in (G) | del (G) | TP |
| TP_0132(-) | - | 153123 - 153124 | 154337 | - | - | del (G) | in (G) | TP*^c^* |
| TP_0135(-) | TPASS_0135(-) | 155746 - 155747 | 156961 | 157004 - 157005 | 157012 | del (C) | in (C) | TP |
| TP_0172(-) | TPASS_0172(-) | 191665 - 191666 | 192818 | 192848 - 192849 | 192858 | del (C) | in (C) | EP |
| TP_0173(-) | TPASS_0173(-) | 192148 | 193300 - 193301 | 193331 | 193340 - 193341 | in (G) | del (G) | GF*^a^* |
| TP_0174(-) | TPASS_0174(-) | 193469 | 194620 - 194621 | 194652 | 194660 - 194661 | in (G) | del (G) | GF |
| TP_0175(-) | TPASS_0175(-) | 193469 | 194620 - 194621 | 194652 | 194660 - 194661 | in (G) | del (G) | GF*^a^* |
| TP_0175(-) | TPASS_0175(-) | 193969 | 195119 - 195120 | 195152 | 195159 - 195160 | in (G) | del (G) | GF |
| TP_0176(-) | TPASS_0176(-) | 194094 - 194095 | 195245 | 195277 - 195278 | 195285 | del (G) | in (G) | GF*^a^* |
| TP_0177(-) | TPASS_0177(-) | 195483 | 196633 - 196634 | 196666 | 196673 - 196674 | in (G) | del (G) | TP |
| - | TPASS_0180(-) | - | - | 199709 - 199710 | 199717 - 199718 | del (GG) | in (GG) | GF |
| TP_0208(+) | TPASS_0208(+) | 215050 - 215051 | 216201 | 216233 - 216234 | 216243 | del (G) | in (G) | TP |
| TP_0217 (+) | TPASS_0217(+) | 221630 - 221631 | 222782 | 222813 - 222814 | 222824 | del (G) | in (G) | AF*^d^* |
| TP_0221(+) | TPASS_0221(+) | 226319 - 226320 | 227472 | 227502 - 227503 | 227514 | del (C) | in (C) | TP |
| TP_r01() | TPASS_r0001() | 230294 - 230295 | 231449 | 231478 - 231479 | 231492 | del (G) | in (G) | - |
| TP_0248(+) | TPASS_0248(+) | 261066 | 262221 - 262222 | 262249 | 262263 - 262264 | in (C) | del (C) | TP*^c^* |
| TP_0263(+) | TPASS_0263(+) | 274268 - 274269 | 295424 | 275451 - 275452 | 275466 | del (G) | in (G) | TP |
| - | TPASS_0279(+) | - | - | 295441 | 295456 - 295457 | in (G) | del (G) | EP*^b^* |
| TP_0284(+) | TPASS_0284(+) | 299184 - 299185 | 300342 | 300367 - 300368 | 300383 | del (A) | in (A) | GF |
| TP_0285(+) | TPASS_0285(+) | 299184 - 299185 | 300342 | 300367 - 300368 | 300383 | del (A) | in (A) | GF*^a^* |
| TP_0286(+) | TPASS_0286(+) | 300588 - 300589 | 301747 | 301771 - 301772 | 301788 | del (G) | in (G) | GF |
| TP_0287(+) | TPASS_0287(+) | 300588 - 300589 | 301747 | 301771 - 301772 | 301788 | del (G) | in (G) | GF*^a^* |
| TP_0288(+) | TPASS_0288(+) | 302114 | 303272 - 303273 | 303297 | 303313 - 303314 | in (G) | del (G) | GF |
| TP_0299(+) | TPASS_0299(+) | 312355 - 312356 | 313514 | 313538 - 313539 | 313555 | del (C) | in (C) | GF*^a^* |
| TP_0318(-) | - | 334673 | 335831 - 335832 | - | - | in (C) | del (C) | TP*^a^* |
| TP_0324(+) | TPASS_0324(+) | 341303 - 341304 | 342462 | 342483 - 342484 | 342501 | del (G) | in (G) | GF |
| TP_0329(+) | TPASS_0329(+) | 351799 | 353959 - 353960 | 352981 | 352998 - 352999 | in (G) | del (G) | TP |
| - | TPASS_0347(+) | - | - | 373240 - 373242 | 373256 - 373257 | in (GGG) | del (GGG) | TP |
| TP_0348(-) | TPASS_0348(-) | 372893 - 372894 | 374052 | 374073 - 374074 | 374088 | del (G) | in (G) | TP |
| TP_0376(-) | TPASS_0376(-) | 401712 - 401713 | 402871 | 402892 - 402893 | 402907 | del (C) | in (C) | TP*^c^* |
| TP_0377(+) | TPASS_0377(+) | 401932 - 401933 | 403093 | 403113 - 403114 | 403129 | del (C) | in (C) | GF |
| TP_0407(+) | TPASS_0407(+) | 431561 | 432720 - 432721 | 432741 | 432753 - 432754 | in (C) | del (C) | TP |
| TP_0415(+) | TPASS_0415(+) | 442642 - 442643 | 443804 | 443822 - 443823 | 443837 | del (G) | in (G) | TP |
| TP_0419(+) | TPASS_0419(+) | 447480 | 448641 - 448642 | 448660 | 448674 - 448675 | in (T) | del (T) | GF |
| TP_0420(+) | TPASS_0420(+) | 447480 | 448641 - 448642 | 448660 | 448674 - 448675 | in (T) | del (T) | GF*^a^* |
| TP_0424(+) | TPASS_0424(+) | 452444 - 452445 | 453607 | 453624 - 453625 | 453640 | del (G) | in (G) | TP |
| TP_0425(+) | TPASS_0425(+) | 452817 | 454980 - 454981 | 453998 | 454013 - 454014 | in (C) | del (C) | EP |
| - | TPASS_0461(+) | - | - | 492420 | 492434 - 492435 | in (C) | del (C) | EP |
| TP_0462(+) | TPASS_0462(+) | 491873 | 493454 - 493455 | 493472 | 493486 - 493487 | in (G) | del (G) | GF |
| TP_0469(-) | - | 495741 - 495742 | 497323 | - | - | del (T) | in (T) | GF |
| TP_0477(-) | TPASS_0477(-) | 506590 - 506591 | 508173 | 508022 - 508023 | 508037 | del (C) | in (C) | TP |
| - | TPASS_0479(-) | - | - | 510907 - 510908 | 510923 | del (C) | in (C) | EP |
| TP_0481(+) | TPASS_0481(+) | 511592 - 511593 | 513176 | 513024 - 513025 | 513041 | del (C) | in (C) | GF |
| TP_0486(-) | - | 517597 | 519181 - 519182 | - | - | in (T) | del (T) | EP |
| TP_0487(-) | - | 520371 - 520372 | 521956 | - | - | del (T) | in (T) | TP |
| - | TPASS_0487(-) | - | - | 521803 - 521804 | 521821 | del (C) | in (C) | TP |
| TP_0491(+) | TPASS_0491(+) | 525990 - 525991 | 527576 | 527422 - 527423 | 527441 | del (G) | in (G) | EP |
| TP_0520(-) | TPASS_0520(-) | 561288 - 561289 | 563875 | 562719 - 562720 | 562739 | del (C) | in (C) | none*^e^* |
| TP_0533(-) | TPASS_0533(-) | 575981 - 575982 | 577569 | 577415 - 577416 | 577436 | del (G) | in (G) | EP |
| TP_0536(-) | TPASS_0536(-) | 578544 - 578545 | 580134 | 579976 - 579977 | 579999 | del (C) | in (C) | EP |
| TP_0536(-) | TPASS_0536(-) | 578593 - 578594 | 580184 | 580025 - 580026 | 580049 | del (C) | in (C) | EP |
| TP_0555(-) | TPASS_0555(-) | 600947 - 600948 | 602539 | 602397 - 602398 | 602422 | del (G) | in (G) | TP |
| TP_0575(+) | TPASS_0575(+) | 624228 - 624229 | 625821 | 625678 - 625679 | 625704 | del (C) | in (C) | AF*^d^* |
| TP_0588(-) | TPASS_0588(-) | 639922 - 639923 | 641516 | 641372 - 641373 | 641399 | del (G) | in (G) | GF*^a^* |
| TP_0594(-) | TPASS_0594(-) | 646342 | 648935 - 648936 | 647792 | 647818 - 647819 | in (C) | del (C) | EP |
| TP_0598(-) | - | 649125 - 649126 | 650719 | - | - | del (G) | in (G) | GF |
| TP_0598(-) | - | 649203 - 649204 | 650798 | - | - | del (A) | in (A) | none |
| TP_0598(-) | - | 649208 - 649209 | 650804 | - | - | del (A) | in (A) | none |
| TP_0598(-) | - | 649225 - 649226 | 650822 | - | - | del (A) | in (A) | none |
| TP_0618(-) | - | 669837 | 671435 - 671436 | - | - | in (C) | del (C) | EP |
| TP_0651(-) | TPASS_0651(-) | 715501 - 715502 | 717099 | 716956 - 716957 | 716986 | del (G) | in (G) | TP*^c^* |
| TP_0703(-) | TPASS_0703(-) | 767896 - 767897 | 769496 | 769344 - 769345 | 769376 | del (C) | in (C) | GF*^a^* |
| TP_0731(+) | TPASS_0731(+) | 797298 - 797299 | 798899 | 798746 - 798747 | 798779 | del (G) | in (G) | TP |
| TP_0762(+) | TPASS_0762(+) | 825078 - 825079 | 826680 | 826526 - 826527 | 826560 | del (C) | in (C) | EP*^a^* |
| TP_0782(-) | TPASS_0782(-) | 848920 | 850522 - 850523 | 850368 | 850402 - 850403 | in (A) | del (A) | GF*^a^* |
| TP_0813(-) | TPASS_0813(-) | 881462 - 881463 | 883065 | 882910 - 882911 | 882945 | del (C) | in (C) | TP |
| TP_0830(+) | TPASS_0830(+) | 897331 - 897332 | 898935 | 898779 - 898780 | 898815 | del (C) | in (C) | TP |
| TP_0856(+) | TPASS_0856(+) | 934065 - 934066 | 935670 | 935513 - 935514 | 935550 | del (C) | in (C) | EP |
| TP_0859(+) | TPASS_0859(+) | 936409 | 938013 - 938014 | 937857 | 937893 - 937894 | in (G) | del (G) | GF |
| TP_0866(-) | TPASS_0866(-) | 946165 - 946166 | 947771 | 947617 - 947618 | 947654 | del (C) | in (C) | AF*^d^* |
| TP_0899(-) | TPASS_0899(-) | 980115 - 980116 | 981724 | 981560 - 981561 | 981660 | del (C) | in (C) | GF |
| TP_0900(-) | TPASS_0900(-) | 980115 | 981724 | 981560 - 981561 | 981660 | del (C) | in (C) | GF*^a^* |
| TP_0922(-) | TPASS_0922(-) | 1000782 - 1000783 | 1002392 - 1002393 | 1002227 - 1002228 | 1002328 - 1002329 | del (GC) | in (GC) | TP*^cf^* |
| TP_0922(-) | TPASS_0922(-) | 1001601 - 1001602 | 1003213 | 1003046 - 1003047 | 1003149 | del (C) | in (C) | TP*^f^* |
| TP_0923(-) | TPASS_0923(-) | 1001727 - 1001728 | 1003340 | 1003172 - 1003173 | 1003276 | del (G) | in (G) | FS*^g^* |
| TP_0923(-) | TPASS_0923(-) | 1001788 - 1001789 | 1003402 - 1003403 | 1003233 - 1003234 | 1003338 - 1003339 | del (GC) | in (GC) | FS*^g^* |
| TP_0928(+) | TPASS_0928(+) | 1009246 - 1009247 | 1010863 | 1010691 - 1010692 | 1010800 | del (G) | in (G) | GF |
| TP_0950(-) | TPASS_0950(-) | 1032252 - 1032253 | 1033871 | 1033697 - 1033698 | 1033808 | del (C) | in (C) | TP*^h^* |
| - | TPASS_0969(-) | - | - | 1055379 | 1055489 - 1055490 | in (C) | del (C) | TP*^c^* |
| TP_0993(-) | TPASS_0993(-) | 1077390 - 1077391 | 1079009 | 1078835 - 1078836 | 1078945 | del (G) | in (G) | EP |
| TP_0995(+) | TPASS_0995(+) | 1080768 - 1080769 | 1082388 | 1082213 - 1082214 | 1082324 | del (G) | in (G) | EP |
| TP_1030(-) | TPASS_1030(-) | 1124003 - 1124004 | 1125624 | 1125634 - 1125635 | 1125746 | del (G) | in (G) | TP |
| TP_1030(-) | - | 1124188 - 1124189 | 1125810 | - | - | del (G) | in (G) | TP |

GF, gene fusion; EP, protein elongation; TP, protein truncation; AF, for authentic frameshift;

*^a^*ORF is not annotated in Nichols/SS14-RS genome due to fusion with another ORF; *^b^*an alternative start codon was predicted; *^c^*ORF is not annotated in the Nichols/SS14-RS genome due to its length below 150 bp limit; *^d^*ORF was annotated as containing authentic frameshift in the original Nichols/SS14 genome, correction of the sequencing error resulted in restoration of the reading frame; *^e^*this ORF contains authentic frameshift; *^f^*error is localized outside the newly annotated ORF and have no effect on the Nichols/SS14-RS protein sequence; *^g^*in-frame indel; *^h^*an alternative gene (TPASS_950a) was predicted in this region
